# Comparative Transcriptome Analysis of the Accessory Sex Gland and Testis from the Chinese Mitten Crab (*Eriocheir sinensis*)

**DOI:** 10.1371/journal.pone.0053915

**Published:** 2013-01-16

**Authors:** Lin He, Hui Jiang, Dandan Cao, Lihua Liu, Songnian Hu, Qun Wang

**Affiliations:** 1 School of Life Science, East China Normal University, Shanghai, China; 2 Key Laboratory of Genome Sciences and Information, Beijing Institute of Genomics, Chinese Academy of Sciences, Beijing, China; Auburn University, United States of America

## Abstract

The accessory sex gland (ASG) is an important component of the male reproductive system, which functions to enhance the fertility of spermatozoa during male reproduction. Certain proteins secreted by the ASG are known to bind to the spermatozoa membrane and affect its function. The ASG gene expression profile in Chinese mitten crab (*Eriocheir sinensis*) has not been extensively studied, and limited genetic research has been conducted on this species. The advent of high-throughput sequencing technologies enables the generation of genomic resources within a short period of time and at minimal cost. In the present study, we performed *de novo* transcriptome sequencing to produce a comprehensive transcript dataset for the ASG of *E. sinensis* using Illumina sequencing technology. This analysis yielded a total of 33,221,284 sequencing reads, including 2.6 Gb of total nucleotides. Reads were assembled into 85,913 contigs (average 218 bp), or 58,567 scaffold sequences (average 292 bp), that identified 37,955 unigenes (average 385 bp). We assembled all unigenes and compared them with the published testis transcriptome from *E. sinensis*. In order to identify which genes may be involved in ASG function, as it pertains to modification of spermatozoa, we compared the ASG and testis transcriptome of *E. sinensis*. Our analysis identified specific genes with both higher and lower tissue expression levels in the two tissues, and the functions of these genes were analyzed to elucidate their potential roles during maturation of spermatozoa. Availability of detailed transcriptome data from ASG and testis in *E. sinensis* can assist our understanding of the molecular mechanisms involved with spermatozoa conservation, transport, maturation and capacitation and potentially acrosome activation.

## Introduction

The product of spermatogenesis is a genetically unique male gamete that can fertilize an ovum and produce offspring. Spermatogenesis and the accumulation of spermatozoa occur in the unique tissues of the testis, in a process that involves a series of intricate, cellular, proliferative and developmental phases. Spermatozoa are not capable of fertilizing an oocyte immediately after completing spermatogenesis and spermiation in the testis, though transport through the accessory sex glands (ASG) changes the activity of spermatozoa [1]. The testis and epididymis are the two male reproductive glands that produce spermatozoa and secrete androgens with the testis being responsible for continuous production of spermatozoa, and the epididymis ensuring production of a heterogeneous sperm population capable of fertilizing an oocyte and also acting as a reservoir for male gametes [2]. In mammals, it is well established that some important sperm attributes are acquired during epididymal transit, including motility, oocyte binding, and penetrating capacity, but there is also evidence that secretions from the ASG influence other aspects of sperm physiology and fertilization [3]. Insects and crustaceans have no additional accessorial glands, and the function of the ASG corresponds with the function of the epididymis in mammals. In most species, sperm maturation studies have focused on secretions from the ASG, and have reported that these secretions are able to enhance fertilizing capacity of sperm collected from the cauda epididymis [4].

As stated above, sperm maturation and fertilizing capacity are not intrinsic to sperm themselves but are acquired during their transit through the epididymis [5]. Post-meiotic haploid spermatids differentiate into mature spermatozoa via highly specialized processes, this modification of spermatozoa can occur in the epididymis or ASG [6]. The ASG is known to have a significant function in mammals, and its secretions contain a variety of bioactive molecules that exert wide-ranging effects on female reproductive activity, they also improve the male’s chances of successful reproduction [7]. In addition, some ASG proteins provide nutritional factors to newly developed spermatozoa, and other yet unidentified factors are capable of inducing a cascade of spermatozoa membrane alterations that exert an influence on spermatozoa vitality [8], physiological state, motility and capacitation [9], as well as fertilization capacity [10]. A delicate reorientation and modification of sperm surface molecules takes place when sperm are activated by capacitation factors. These surface changes are probably required to enable the sperm to bind to the extracellular matrix of the oocyte (the zona pellucida, ZP) [11]. For example, sperm surface coating protein that normally prevent adhesion are lost during transit of sperm in the uterus and are recoated in the oviduct. The surface of the sperm cell may also be modified by the oviduct epithelium that adsorbs proteins from the sperm surface and also secretes glycoproteins with an unknown function in sperm–ZP binding [12].

The Chinese mitten crab (*Eriocheir sinensis*) (Henri Milne Edwards 1854) is one of the most important aquaculture species in China and has high commercial value as a food source [13]. *E. sinensis* is a catadromous crustacean with a life-span of about two years. During its complex life cycle, the crab spends most of its life in rivers and lakes [14]. Adults migrate downstream towards estuarine waters, where they reach maturity and mate from November to March before moving into high salinity regions in estuaries where they release the larvae during early spring [15]. This species reproduces only once and dies shortly afterwards. Relative to mammals, *E. sinensis* require more complex environments to induce mating and spawning, and unique regulatory mechanisms are involved in crustacean reproduction. Sexual precocity has been reported in cultured Chinese *E. sinensis* populations since development of their intensive aquaculture in the early 1980s [16]. Precocious crabs mature and die prematurely at a small size, where this occurs it can lead to catastrophic losses for farmers and this problem seriously impacts development of crab aquaculture. The molecular mechanisms underlying *E. sinensis* sexual precocity remain unclear. As a consequence, genetic mechanisms involved in growth, reproduction and immune response of *E. sinensis* are currently an active research area for this economically important species.

Recently, the focus of *E. sinensis* research in reproductive and developmental biology has shifted from histological and biochemical analyses to genetic and molecular studies [17]. In this regard, genes crucial for reproduction and development need to be identified and their regulatory mechanisms elucidated. Transcriptome sequencing yields a subset of genes from the genome that are functionally active in selected tissues and species of interest. In nonstandard model organisms where genomic resources are lacking, such as a fully sequenced genome, obtaining a transcriptome is an effective way to evaluate gene expression and to perform comparative studies at the whole genome level [18]. In order to study gene expression profiles during spermatogenesis, we previously performed *de novo* transcriptome sequencing to produce a comprehensive transcript dataset for *E. sinensis* testis, that produced 25,698,778 sequencing reads corresponding with 2.31 Gb of total nucleotides. Reads were assembled into 342,753 contigs or 141,861 scaffold sequences, that identified 96,311 unigenes [19]. In the above mentioned study, we identified several sperm membrane proteins, that may be modified by ASG proteins during maturation, which we later identified as ASG proteins involved in spermatophore rupture [20]. In a continuation of our previous studies, we have performed a *de novo* transcriptome analysis for the *E. sinensis* ASG, and present a comparative analysis of the transcriptome for both the ASG and testis in *E. sinensis* in order to elucidate ASG function in sperm maturation. The analysis was based on construction of annotated ASG and testis transcriptome libraries by *de novo* assembly of short raw reads generated by high-throughput technology (Illumina Solexa sequencing) without genomic sequence information. We believe global approaches of this type will pave the way to allow development of a more complete understanding of the complex gene and protein networks that drive the biological and reproductive processes of spermatogenesis. The goal of this research is to provide a general overview of the potential molecular mechanisms that are involved in *E. sinensis* reproduction and to find key genes or pathways that function in the process of fertilization and spermatogenesis. Furthermore, we hope to provide fundamental and significant information about the sperm maturation process during transport through the ASG in *E. sinensis*, and elucidate sperm modification mechanisms during the acrosome reaction and sperm-oocyte interactions.

## Materials and Methods

### Tissue Sampling, cDNA Library Creation, and Sequencing

All animal investigations were carried out according to Animal Care and Use of Science and Technology guidelines. Healthy, sexually mature, male mitten crabs (*E. sinensis*, weighing 150 to 200 g) that had reached the stage of rapid ASG development were obtained from a commercial crab farm (Caojing Town special aquaculture farm in Jinshan District) near Shanghai, China between October and December in 2010. Male crabs were dissected on ice, the ASGs were removed immediately and tissues were flash frozen in liquid nitrogen. ASG tissues from three different individuals were taken on three occasions, and the nine pairs of ASG tissue were pooled as a single sample for RNA extraction. Total RNA was isolated using TRIzol reagent (Invitrogen, Shanghai, China). The RNA integrity score and quantity were determined using an Agilent 2100 Bioanalyzer (Agilent, Shanghai, China) before cDNA synthesis. RNA extraction, cDNA synthesis, cDNA library normalization, and Illumina sequencing were performed according to published methods [19].

### Transcriptome Assembly

**Figure 1:**
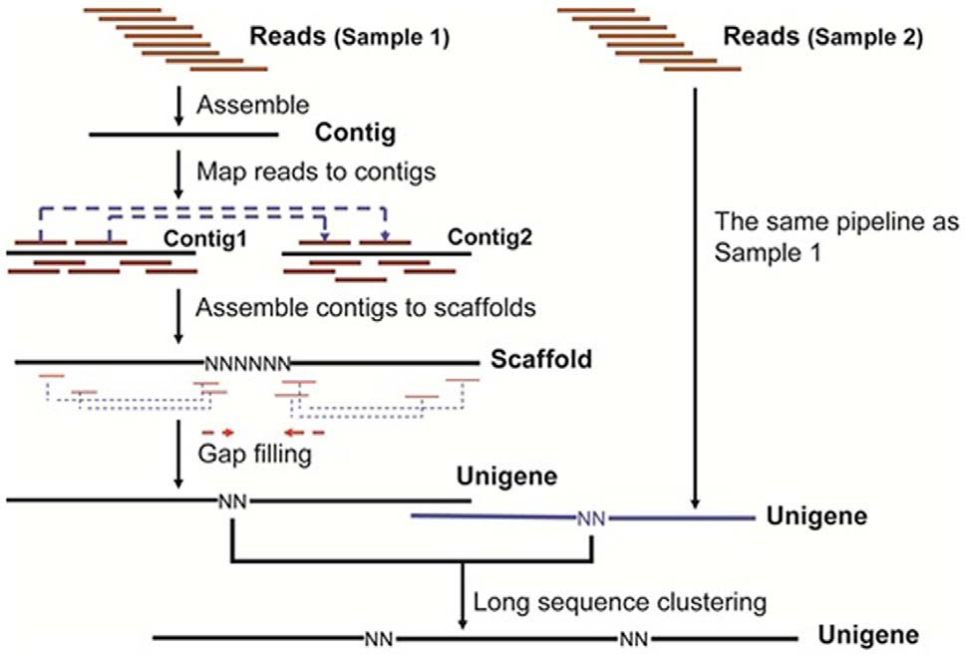
Scheme showing the assembly of unigenes from ASG and testis in *E. sinensis*.

Transcriptome *de novo* assembly was carried out with the short read assembling program SOAPdenovo-v1.03 [21]. All subsequent analyses were based on clean reads. Reads with certain lengths of overlap and no uncalled bases (N) were combined as contigs to form longer fragments. Contigs were then connected using N to represent the unknown sequence between each pair of contigs to form scaffolds. Paired-end reads were used for gap filling of scaffolds to obtain sequences with the smallest number of N’s. These sequences were defined as unigenes. In the final step, Blastx alignments (E-value <10^−5^) between unigenes and sequences in protein databases, including the National Center for Biotechnology Information (NCBI) non-redundant (nr) database, Swiss-Prot, Kyoto Encyclopedia of Genes and Genomes (KEGG; http://www.genome.jp/kegg/) and Clusters of Orthologous Groups (COG) were performed to identify the sequence direction of unigenes. If results of different databases were conflicting, a priority order of alignments from the nr, Swiss-Prot, KEGG and COG databases was followed to decide the sequence direction. When a unigene happened to be unaligned to any sequence in the above databases, the software program ESTScan [22] was used to define the sequence direction. For unigenes with determined sequence directions, we identified their sequences from the 5' to 3' end and for those with undetermined directions, we provided their sequence based on the assembly software. When multiple samples from the same species are sequenced, unigenes from each sample's assembly can be further processed for sequence splicing and removal of redundancy with sequence clustering software to acquire the longest reads of nr unigenes (Fig. 1).

### Homology Searches and Functional Unigene Annotation

Annotation provides information on expression and function of a unigene. In our functional annotation, unigene sequences were first aligned using Blastx to the nr, Swiss-Prot, KEGG and COG protein databases (E-value <10^−5^), to retrieve proteins with the highest sequence similarity to *E. sinensis* unigenes along with their protein functional annotations. Homology searches were carried out by query of the NCBI nr protein database using the Blastx algorithm (E-value <10^−5^) [23]. After nr annotation, we used the Blast2GO program [24] to obtain Gene Ontology (GO) annotations, and WEGO software [25] was used to perform GO functional classification of all unigenes in order to understand the distribution of gene functions at the macro level.

Using EC (Enzyme Commission number) terms, biochemical pathway information was generated by downloading relevant maps from the KEGG database [26]. This database contains systematic analysis of inner-cell metabolic pathways and functions of individual gene products. Here we identified the biological pathways that were active in *E. sinensis* ASG and assessed up or down regulation of key genes involved in the relevant pathways. After obtaining the KEGG pathway annotations, unigenes were aligned to the COG database to predict and classify potential functions based on known orthologous gene products. Every protein in COG is assumed to evolve from an ancestor protein, and the whole database is built on coding proteins with complete genomes as well as systematic evolutionary relationships among bacteria, algae and eukaryotic organisms [27].

### Unigene Expression Difference Analysis

Unigene expression was calculated using the reads per kb per million reads method (RPKM), for which the formula is shown below:
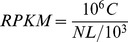
Where RPKM is the expression of unigene A, and C is the number of reads that uniquely aligned to unigene A. N is the total number of reads that uniquely aligned to all unigenes, and L is the base number in the CDS of unigene A. The RPKM method is able to eliminate the influence of different gene length and sequencing level on the calculation of gene expression. Therefore the calculated gene expression level can be used directly for comparing difference in gene expression between samples [28].

### Data Deposition

*De novo* assembly sequence data from *E. sinensis* were deposited in the National Center for Biotechnology Information (NCBI, USA, http://www.ncbi.nlm.nih.gov/), while *de novo* assembly of sequence data from the ASG and testis in *E. sinensis* were deposited in the Transcriptome Shotgun Assembly (TSA) database with accession numbers KA660105–KA728674.

## Results

### General Features of the ASG Transcriptome in *E. sinensis*

**Figure 2:**
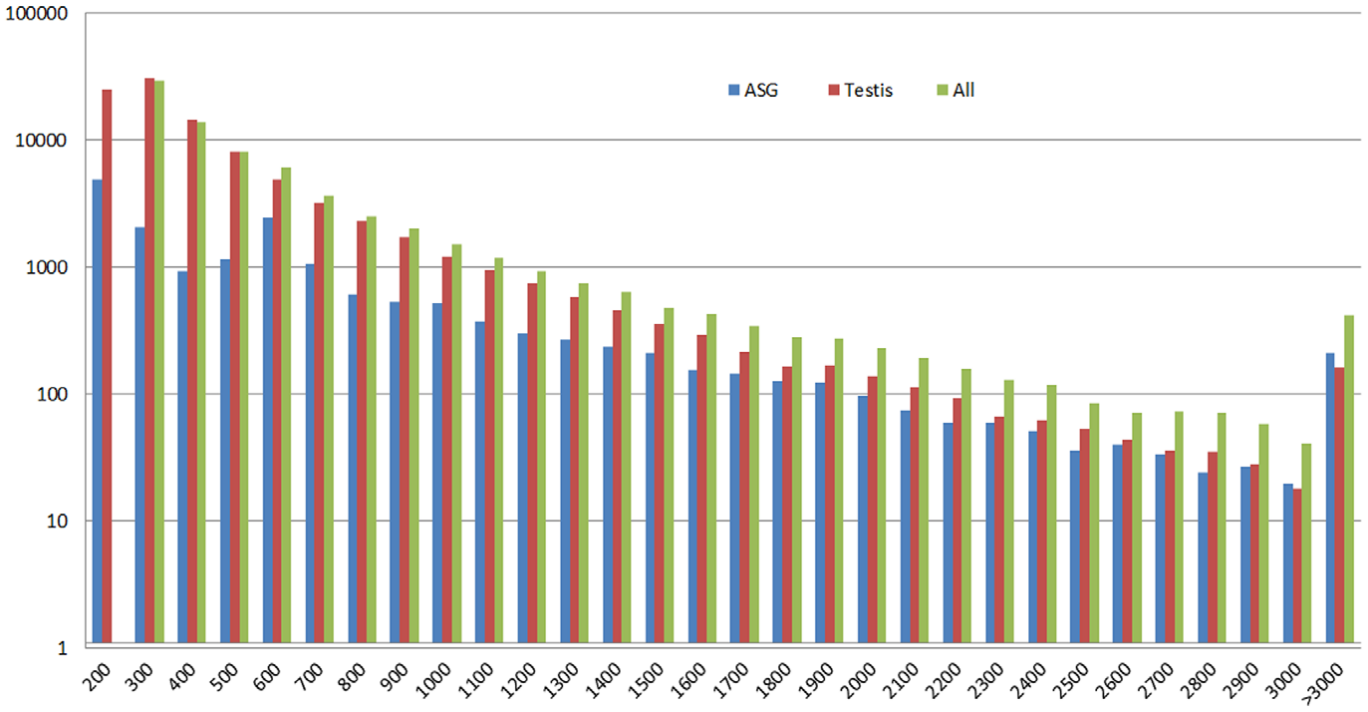
Assembly quality statistics of the ASG, testis and all unigenes from Illumina sequencing. The length distribution of *de novo* assemblies of unigenes are shown (X-axis indicates the sequence size (nt), and the Y-axis indicates the number of assembled unigenes).

Illumina high-throughput second generation sequencing produced 33,221,284 clean reads representing a total of 2,657,702,720 (2.66 Gb) nucleotides. Average read size, Q20 percentage and GC content were 90 bp, 91.06%, and 55.19%, respectively. From these short reads, 85,913 contigs were assembled, with a median length of 218 bp. From the contigs, 58,567 scaffolds were constructed using SOAPdenovo, with a median length of 292 bp, and 37,955 unigenes were obtained with a median length of 385 bp (Table 1). The quality of Illumina short read sequence assemblies results are shown in Figure 2.

**Table 1:** Summary of transcriptomes from the accessory sex gland (ASG) and testis (T) in *E. sinensis*.

	ASG	T	ASG & T
Total Reads	33,221,284	25,698,778	−
Total base pair(bp)	2,657,702,720	2,312,890,020	−
Q20 percentage	91.06%	91.3%	−
N percentage	0.15%	0.01%	−
GC percentage	55.19%	49.17%	−
Total number of contigs	85,913	342,753	−
Mean length of contigs (bp)	218	191	−
Total number of scaffolds	58,567	141,861	−
Mean length of scaffolds (bp)	292	300	−
The number of unigenes	37,955	96311	−
Mean length of unigenes	385	382	−
The number of all-unigenes	−	−	74049
Mean length of all-unigenes	−	−	512

### Unigene Annotation and GO Assignment

Functional annotation consisted of protein functional annotation, pathway annotation, GO assignments and COG functional annotation. Distinct gene sequence analysis identified 27,541 unigene annotations (37.2% of all unigenes) above the preset cut-off value; similarly, 6,350 (8.6%) unigenes were annotated via ESTscan analysis. Based on similarity searches with known proteins, 33,891 unigenes were annotated based on having a Blast hit in the nr database or ESTscan results (Table S1). Since no genome or EST information existed previously for *Eriocheir* species, 54.2% of the unigenes could not be matched to known genes, though it is likely that many of the genes of unknown function and/or unknown protein product would share common functions with known genes within the same cluster in the GO clustering analysis. Annotation analysis was used to provide information on gene expression and functional annotation of all unigenes from ASG and testis from *E. sinensis* resulted in 74,049 distinct events (Table 1). This number does not necessarily reflect the real transcriptome complexity, as many of the assembled sequences may represent distinct non-overlapping regions of the same transcripts. Thus, the final number of unique transcripts covered by our data would probably be lower.

**Figure 3:**
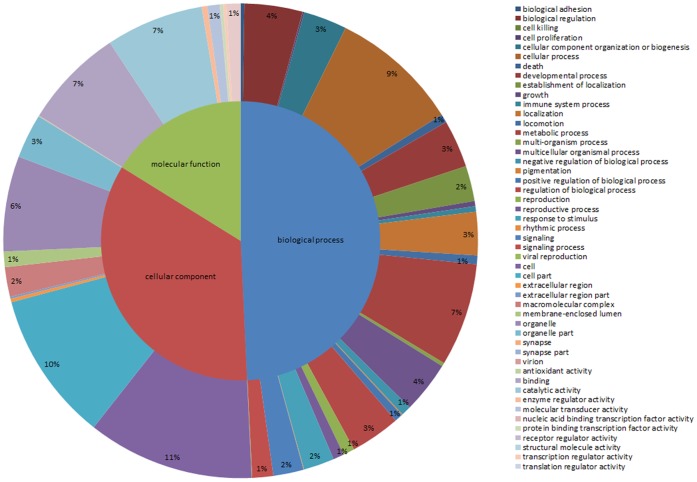
GO classification of all unigenes from ASG and testis in *E. sinensis*.

GO assignments were used to classify the functions of the predicted genes. Based on sequence homology, sequences can be categorized into 43 functional groups; the best hits from this query were extracted for GO classification using Uniprot2GO; each sequence was assigned at least one GO term. Second-level GO terms were used to classify the sequences in terms of their involvement as cellular components, in molecular functions, and in biological processes (Fig. 3). In total, 44,144 unigenes were clustered in three assignments; 15,261 were categorized as “Cellular Component” (34.6%), 21,745 as “Biological Process” (49.3%) and 7,138 as “Molecular Function” (16.2%).

**Figure 4:**
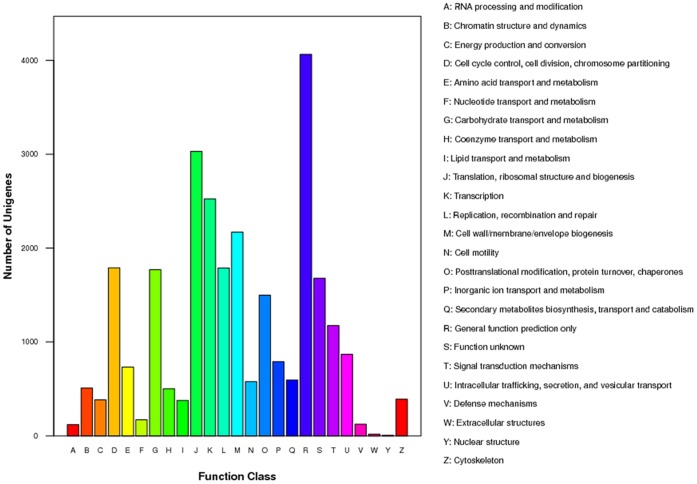
COG classification of all unigenes from ASG and testis in *E. sinensis*.

Using COG functional annotation, 27,657 unigenes were assigned into 25 function classes. 4,062 unigenes (14.7%) were assigned into “General function prediction only”, 3,030 unigenes were annotated into “Translation, ribosomal structure and biogenesis” and 2,524 unigenes were related to “Transcription”. The most abundantly represented biological processes were “Cell wall/membrane/envelope biogenesis”, “Cell cycle control, cell division, chromosome partitioning” and “Replication, recombination and repair” which comprised 2,171, 1,790 and 1,788 unigenes respectively, of the biological process sequences (Fig. 4).

### KEGG Pathway Assignment

We mapped the 17,645 annotated sequences to the reference canonical pathways in the KEGG database to identify the biological pathways involved. A total of 17,645 unigenes were associated with 225 predicted KEGG metabolic pathways, and the number of different expressed genes (DEG) with pathway annotation was 11,962 (Table S3). The top two most prominent pathways (metabolic pathways and regulation of actin cytoskeleton) included over 1,510 unigenes. The most important pathways that may be relevant to spermatogenesis or reproduction included regulation of actin cytoskeleton (1,146 unigenes), DNA replication (90 unigenes), splicesome (1,007 unigenes), RNA polymerase (234 unigenes), Mismatch repair (60 unigenes), purine metabolism (468 unigenes), adherens junction (769 unigenes), cell cycle (272 unigenes), Fc gamma R-mediated phagocytosis (708 unigenes), pyrimidine metabolism (357 unigenes) and other anti-hyperthermia stress and anti-oxidative stress pathways or gene families such as the proteasome (53 unigenes). The top 30 pathways with highest DEGs genes are shown in Table 2. These predicted pathways are likely to be useful in future investigations that focus on their functions in *E. sinensis*. Using KEGG, 1,704 unigenes (14.99%) were included in basic metabolism process specific pathways; most of these were involved in carbohydrate, energy, and amino acid metabolism.

**Table 2:** All unigenes KEGG metabolic pathway analysis in *E. sinensis*.

No.	Pathway	DEGs genes with pathway annotation (11,962)	All genes with pathway annotation (17,645)	P value	Q value	Pathway ID
1	Vibrio cholerae infection	657 (5.49%)	851 (4.82%)	3.70E-10	8.33E-08	ko05110
2	Phototransduction	505 (4.22%)	659 (3.73%)	2.36E-07	2.66E-05	ko04744
3	Olfactory transduction	506 (4.23%)	666 (3.77%)	1.47E-06	1.10E-04	ko04740
4	DNA replication	80 (0.67%)	90 (0.51%)	2.73E-06	1.54E-04	ko03030
5	Pyrimidine metabolism	279 (2.33%)	357 (2.02%)	8.20E-06	3.69E-04	ko00240
6	Amoebiasis	710 (5.94%)	962 (5.45%)	1.77E-05	6.66E-04	ko05146
7	Spliceosome	739 (6.18%)	1007 (5.71%)	4.16E-05	1.22E-03	ko03040
8	RNA polymerase	186 (1.55%)	234 (1.33%)	4.35E-05	1.22E-03	ko03020
9	Amyotrophic lateral sclerosis (ALS)	217 (1.81%)	277 (1.57%)	6.09E-05	1.52E-03	ko05014
10	Homologous recombination	46 (0.38%)	51 (0.29%)	0.000174216	3.92E-03	ko03440
11	Mismatch repair	53 (0.44%)	60 (0.34%)	0.000207332	4.24E-03	ko03430
12	Purine metabolism	349 (2.92%)	468 (2.65%)	0.000714386	1.34E-02	ko00230
13	Base excision repair	72 (0.6%)	87 (0.49%)	0.001272401	2.20E-02	ko03410
14	Nucleotide excision repair	89 (0.74%)	110 (0.62%)	0.00151106	2.43E-02	ko03420
15	Regulation of actin cytoskeleton	821 (6.86%)	1146 (6.49%)	0.002011545	3.02E-02	ko04810
16	Pathogenic Escherichia coli infection	455 (3.8%)	624 (3.54%)	0.002713139	3.82E-02	ko05130
17	Neuroactive ligand-receptor interaction	183 (1.53%)	241 (1.37%)	0.003346124	4.43E-02	ko04080
18	Proteasome	45 (0.38%)	53 (0.3%)	0.003901236	4.88E-02	ko03050
19	Cardiac muscle contraction	167 (1.4%)	220 (1.25%)	0.005031368	5.96E-02	ko04260
20	Adherens junction	552 (4.61%)	769 (4.36%)	0.008131791	9.15E-02	ko04520
21	Bacterial invasion of epithelial cells	477 (3.99%)	662 (3.75%)	0.008830244	9.46E-02	ko05100
22	Fc gamma R-mediated phagocytosis	508 (4.25%)	708 (4.01%)	0.01130496	1.16E-01	ko04666
23	Shigellosis	477 (3.99%)	666 (3.77%)	0.01654301	1.62E-01	ko05131
24	Cell cycle	200 (1.67%)	272 (1.54%)	0.02275106	2.13E-01	ko04110
25	Viral myocarditis	150 (1.25%)	202 (1.14%)	0.02689615	2.42E-01	ko05416
26	Huntington’s disease	404 (3.38%)	566 (3.21%)	0.03420199	2.96E-01	ko05016
27	SNARE interactions in vesicular transport	36 (0.3%)	45 (0.26%)	0.0512175	4.12E-01	ko04130
28	Staphylococcus aureus infection	36 (0.3%)	45 (0.26%)	0.0512175	4.12E-01	ko05150
29	Dorso-ventral axis formation	225 (1.88%)	312 (1.77%)	0.05487221	4.26E-01	ko04320
30	Riboflavin metabolism	25 (0.21%)	31 (0.18%)	0.08619657	6.46E-01	ko00740

### Tissue-specific Analysis for Differentially Expressed Genes

With regard to tissue specific analysis of differentially regulated genes, numerous genes crucial for reproduction and development were identified, including fertilin, serine proteinase inhibitor, Sperm antigen P26h, Sperm protamine and bovine seminal plasma protein BSP (Table 3). Identification of these essential genes and their regulatory mechanisms provided new understanding about the complex processes of reproduction and development. We believe information gained about these genes in *E. Sinensis* can be applied to this species to improve industrial aquaculture.

**Table 3:** The reproduction-related unigenes identified in the accessory sex gland (ASG) and testis (T) transcriptomes during the sexual maturation stage in *E. sinensis*.

Unigene No.	Unigene name of top BLASTX hit (Accession no; species)	Length (bp)	E-value	ASG RPKM	Testis RPKM	Log2(Testis RPKM/ASG RPKM)
**Sperm antigen P26h (L-xylulose reductase)**						
Unigene4288_All	L-xylulose reductase (gi|229365856|gb|ACQ57908.1|;*Anoplopoma fimbria*)	989	5.00E-82	27.5693	19.8661	−0.4728
**BSP (bovine seminal plasma protein)**						
Unigene64588_All	surface antigen BspA-like (gi|123302396|ref|XP_001291104.1|;*Trichomonas vaginalis* G3)	384	8.00E-06	0	6.192	12.5962
Unigene69768_All	surface antigen BspA-like (gi|123302396|ref|XP_001291104.1|;*Trichomonas vaginalis* G3)	526	3.00E-07	0	4.7583	12.2162
**Fertilin**						
Unigene17270_All	similar to fertilin alpha-I (gi|126324185|ref|XP_001371111.1|;*Monodelphis domestica*)	625	5.00E-06	0	16.619	14.0206
Unigene18613_All	similar to fertilin alpha-I (gi|126324185|ref|XP_001371111.1|;*Monodelphis domestica*)	580	9.00E-09	0.8666	8.1991	3.242
Unigene62731_All	similar to fertilin alpha-I(gi|126324185|ref|XP_001371111.1|;*Monodelphis domestica*)	356	2.00E-09	0	9.4912	13.2124
Unigene71804_All	similar to fertilin alpha-I (gi|126324185|ref|XP_001371111.1|;*Monodelphis domestica*)	675	2.00E-11	0	17.639	14.1065
Unigene27136_All	fertilin alpha subunit-like(gi|291406979|ref|XP_002719798.1|;*Oryctolagus cuniculus*)	339	1.00E-06	2.2239	7.3831	1.7311
**Immunoglobulin**						
Unigene2775_All	leucine-rich repeats and immunoglobulin-like domains 2-like (gi|291228204|ref|XP_002734069.1|;*Saccoglossus kowalevskii*)	1394	6.00E-06	0	4.0398	22.0699
Unigene21708_All	Leucine-rich repeats and immunoglobulin-like domains protein 3 (gi|307207257|gb|EFN85034.1|;*Harpegnathos saltator*)	1627	6.00E-59	0	25.2287	14.6228
Unigene24011_All	immunoglobulin superfamily DCC subclass member 4 (gi|292616070|ref|XP_002662886.1|;Danio rerio)	512	4.00E-09	3.9266	10.5101	1.4204
Unigene28249_All	immunoglobulin mu binding protein 2 (gi|296471357|gb|DAA13472.1|;*Bos Taurus*)	442	2.00E-23	0.5886	6.2289	3.4535
Unigene67254_All	Leucine-rich repeats and immunoglobulin-like domains protein 3 (gi|307207257|gb|EFN85034.1|;*Harpegnathos saltator*)	440	9.00E-21	0.2856	13.0832	5.5176
Unigene67826_All	Leucine-rich repeats and immunoglobulin-like domains protein 3 (gi|307205378|gb|EFN83719.1|;*Harpegnathos saltator*)	455	1.00E-11	0	39.6059	15.2734
**serine proteinase inhibitor**						
Unigene12520_All	serine proteinase inhibitor 6(gi|288188852|gb|ADC42876.1|;*Penaeus monodon*)	796	4.00E-36	0.3157	0.4716	0.579
Unigene13284_All	serine proteinase inhibitor 6(gi|288188852|gb|ADC42876.1|;*Penaeus monodon*)	441	1.00E-16	57.8388	33.769	−0.7763
Unigene13350_All	serine proteinase inhibitor (gi|33590491|gb|AAQ22771.1|;*Procambarus clarkii*)	653	3.00E-38	15.2011	0.9582	−3.9877
Unigene14659_All	serine proteinase inhibitor (gi|33590491|gb|AAQ22771.1|;*Procambarus clarkii*)	327	4.00E−18	3.8425	11.8638	1.6264
Unigene1959_All	serine proteinase inhibitor 6 (gi|288188852|gb|ADC42876.1|;*Penaeus monodon*)	1229	3.00E-79	5.6231	10.3862	0.8852
Unigene24440_All	serine proteinase inhibitor (gi|33590491|gb|AAQ22771.1|;*Procambarus clarkii*)	654	2.00E-21	0.9606	23.5362	4.6148
Unigene31267_All	serine proteinase inhibitor (gi|33590491|gb|AAQ22771.1|;*Procambarus clarkii*)	659	4.00E-26	0.3813	12.3435	5.0167
Unigene32663_All	Kazal-type serine proteinase inhibitor 1 [gi|219809644|gb|ACL36280.1|;*Fenneropenaeus chinensis*)	1828	2.00E-64	0.6186	28.616	5.5317
Unigene3605_All	serine proteinase inhibitor (gi|33590491|gb|AAQ22771.1|*Procambarus clarkii*)	531	2.00E-16	23.3501	10.8411	−0.9085
Unigene5046_All	serine proteinase inhibitor 6 (gi|288188852|gb|ADC42876.1|;*Penaeus monodon*)	316	7.00E−11	338.7773	116.0352	−1.5458
Unigene67995_All	serine proteinase inhibitor 6 (gi|288188852|gb|ADC42876.1|;*Penaeus monodon*)	459	8.00E-17	19.436	12.5416	−0.632
**trypsin-like serine protease**						
Unigene43562_All	trypsin-like serine protease (gi|254680853|gb|ACT78700.1|;*Eriocheir sinensis*)	233	3.00E-39	2.6963	2.6855	−0.0058
Unigene49929_All	trypsin-like serine protease (gi|124518462|gb|ABN13876.1|;*Locusta migratoria manilensis*)	261	3.00E-07	0	7.6716	12.9053
Unigene50348_All	trypsin-like serine protease (gi|254680853|gb|ACT78700.1|*Eriocheir sinensis*)	263	6.00E-25	1.4333	7.1375	2.3161
**Dehydrogenase**						
Unigene7579_All	sorbitol dehydrogenase (gi|58332224|ref|NP_001011264.1|;*Xenopus (Silurana) tropicalis*)	950	1.00E-113	12.0359	8.6942	−0.4692
Unigene7617_All	Dihydrolipoyllysine-residue acetyltransferase component of pyruvate dehydrogenase complex, mitochondrial (gi|307183310|gb|EFN70179.1|;*Camponotus floridanus*)	422	2.00E-33	5.0617	16.0137	1.6616
Unigene7691_All	D-beta-hydroxybutyrate dehydrogenase, mitochondrial (gi|147899736|ref|NP_001082978.1|;*Danio rerio*)	667	5.00E-50	21.287	8.8182	−1.2714
Unigene7769_All	Zinc-type alcohol dehydrogenase-like protein C1773.06c (gi|307174541|gb|EFN64991.1|;*Camponotus floridanus*)	4041	3.00E-99	8.0222	11.6751	0.5414
Unigene8005_All	glucose-6-phosphate dehydrogenase isoform B (gi|61394184|gb|AAX45785.1|;*Ips typographus*)	1730	0	14.526	24.9564	0.7808
Unigene8007_All	similar to aldehyde dehydrogenase family 6, subfamily A1 (gi|224051481|ref|XP_002199925.1|;*Taeniopygia guttata*)	2071	1.00E-174	40.5889	49.7312	0.2931
Unigene8101_All	isovaleryl coenzyme A dehydrogenase (gi|209571446|ref|NP_001129356.1|;*Bombyx mori*)	625	6.00E-95	6.8353	7.4085	0.1162
Unigene8139_All	glyceraldehyde 3-phosphate dehydrogenase (gi|296785436|gb|ADH43624.1|;*Eriocheir sinensis*)	1401	0	204.8423	351.2243	0.7779
Unigene8170_All	similar to NADH dehydrogenase (ubiquinone) Fe-S protein 1 isoform 1 and 2 (gi|72133227|ref|XP_780124.1|;*Strongylocentrotus purpuratus*)	2322	0	15.0975	17.5697	0.2188
Unigene819_All	NADH dehydrogenase flavoprotein 2, mitochondrial (gi|170068588|ref|XP_001868925.1|;*Culex quinquefasciatus*)	512	2.00E-71	11.2888	27.864	1.3035
Unigene8242_All	PREDICTED: similar to isocitrate dehydrogenase (gi|189237290|ref|XP_974070.2|;*Tribolium castaneum*)	786	1.00E-101	5.9148	15.7624	1.4141
Unigene8243_All	15-hydroxyprostaglandin dehydrogenase [NAD+] (gi|307184287|gb|EFN70745.1|;*Camponotus floridanus*)	1168	6.00E-52	5.4864	12.1072	1.1419
Unigene8353_All	NADH dehydrogenase subunit 1 (gi|63025123|ref|YP_232831.1|;*Eriocheir sinensis*)	685	1.00E-96	2.3846	339.9891	7.1556
Unigene8999_All	hydroxyacyl dehydrogenase (gi|157122882|ref|XP_001659938.1|;*Aedes aegypti*)	818	5.00E-64	5.5298	7.3434	0.4092
**Glycosyl-phosphatidyl inositol**						
Unigene64835_All	PREDICTED: similar to glycosyl-phosphatidyl inositol-specific phospholipase C (gi|91088447|ref|XP_968769.1|;*Tribolium castaneum*)	388	1.00E-18	0	11.2887	13.4626
Unigene71361_All	Glycosyl-phosphatidyl inositol anchor attachment 1 protein (gi|307188892|gb|EFN73441.1|;*Camponotus floridanus*)	621	7.00E-24	3.8444	11.8897	1.6289
**Estrogen receptor**						
Unigene67401_All	ligand-independent activating molecule for estrogen receptor-like (gi|293351305|ref|XP_002727750.1|;*Rattus norvegicus*)	444	5.00E-06	0	4.7915	12.2263
Unigene7117_All	Breast cancer anti-estrogen resistance protein 1 (gi|307196700|gb|EFN78159.1|;*Harpegnathos saltator*)	3294	1.00E-102	22.4679	13.335	−0.7526
Unigene73158_All	estrogen sulfotransferase-like (gi|110764250|ref|XP_394850.3|;*Apis mellifera*)	903	1.00E-09	0.974	25.7771	4.726
Unigene11682_All	estrogen sulfotransferase-like (gi|110764250|ref|XP_394850.3|;*Apis mellifera*)	1682	3.00E-47	9.2631	25.1478	1.4409
Unigene23879_All	ligand-independent activating molecule for estrogen receptor-like (gi|293351305|ref|XP_002727750.1|;*Rattus norvegicus*)	439	1.00E-06	1.1449	7.1266	2.638
Unigene30622_All	similar to Deoxynucleotidyltransferase terminal-interacting protein 2 (Terminal deoxynucleotidyltransferase-interacting factor 2) (TdT-interacting factor 2) (Estrogen receptor-binding protein) (LPTS-interacting protein 2) (LPTS-RP2) (gi|189235505|ref|XP_969663.2|;*Tribolium castaneum*)	1317	6.00E-38	0.9541	22.4252	4.5548
Unigene47381_All	estrogen-related receptor beta like 1-like (gi|291225239|ref|XP_002732609.1|;*Saccoglossus kowalevskii*)	249	7.00E-18	0	7.0362	12.7806
Unigene55150_All	ras-related and estrogen-regulated growth inhibitor-like (gi|296210885|ref|XP_002752248.1|;*Callithrix jacchus*)	288	5.00E-14	1.7451	3.4762	0.9942
Unigene59552_All	Ras-related and estrogen-regulated growth inhibitor (gi|223649254|gb|ACN11385.1|;*Salmo salar*)	322	9.00E-16	3.9022	4.2751	0.1317
Unigene61508_All	estrogen-related receptor beta like 1-like (gi|291225239|ref|XP_002732609.1|;*Saccoglossus kowalevskii*)	341	3.00E-20	7.0333	6.6058	3.3921
**Epididymal secretory glutathione peroxidase**						
Unigene15860_All	epididymal secretory glutathione peroxidase precursor (gi|47523090|ref|NP_999051.1|;*Sus scrofa*)	381	4.00E-13	0	4.27	12.06
Unigene27168_All	glutathione peroxidase (gi|171189511|gb|ACB42236.1|;*Metapenaeus ensis*)	413	2.00E-39	2.7381	7.8783	1.5247
Unigene27684_All	glutathione peroxidase 7 (gi|148236625|ref|NP_001088904.1|;*Xenopus laevis*)	879	6.00E-40	3.5737	19.6471	2.4588
Unigene62805_All	selenium-dependent glutathione peroxidase (gi|222875570|gb|ACM68948.1|;*Macrobrachium rosenbergii*)	357	7.00E-29	2.4637	2.1033	−0.2282
Unigene63410_All	phospholipid-hydroperoxide glutathione peroxidase (gi|164608818|gb|ABY62740.1|;*Artemia franciscana*)	365	2.00E-49	11.0159	128.2295	3.5411
Unigene65933_All	glutathione peroxidase (gi|171189511|gb|ACB42236.1|;*Metapenaeus ensis*)	409	3.00E-12	0	7.9553	12.9577
Unigene7003_All	phospholipid-hydroperoxide glutathione peroxidase (gi|164608818|gb|ABY62740.1|;*Artemia franciscana*)	459	9.00E-29	7.3912	94.8802	3.6822
Unigene7010_All	dehydrogenase/reductase SDR family member 12-like (gi|292611020|ref|XP_002660947.1|*Danio rerio*)	663	2.00E-64	70.3108	41.1483	−0.7729
Unigene70401_All	FAD-dependent oxidoreductase domain containing 1 (gi|156717942|ref|NP_001096513.1|;*Xenopus (Silurana) tropicalis*)	561	9.00E−48	0	13.6074	13.7321
Unigene71193_All	NADH-Ubiquinone oxidoreductase AGGG subunit (gi|242017690|ref|XP_002429320.1|;*Pediculus humanus corporis*)	614	2.00E-16	3.0696	14.8787	2.2771
**Sperm protamine**						
Unigene13877_All	Sperm protamine P1(sp|P83211|HSP1_MURBR; *Murex brandaris*)	239	7.00E-07	3.6080	14.1376	1.9417
Unigene23278_All	Sperm protamine P2(sp|P83212|HSP2_MURBR; *Murex brandaris*)	297	2.00E-06	1.6923	11.3767	2.749
Unigene29647_All	Sperm protamine P2(sp|P83212|HSP2_MURBR; *Murex brandaris*)	440	6.00E-10	0	15.0741	13.8798
Unigene59678_All	Sperm protamine P1(sp|P83211|HSP1_MURBR; *Murex brandaris*)	322	8.00E−08	0	60607	12.6898
**Angiotensin converting enzyme(ACE)**						
Unigene7164_All	angiotensin converting enzyme (gi|224028155|emb|CAX48990.1|;*Pontastacus leptodactylus*)	3013	2.00E-161	6.1303	4.4027	−0.4776
Unigene21069_All	angiotensin converting enzyme (gi|224028155|emb|CAX48990.1|;*Pontastacus leptodactylus*)	2046	0	5.2201	16.0864	1.6237
Unigene50101_All	angiotensin converting enzyme (gi|224028155|emb|CAX48990.1|;*Pontastacus leptodactylus*)	261	6.00E-19	0	5.2743	12.3648
Unigene60380_All	PREDICTED: similar to angiotensin converting enzyme (gi|198420807|ref|XP_002123029.1|;*Ciona intestinalis*)	329	2.00E-18	2.6734	5.3253	0.9942

**Figure 5:**
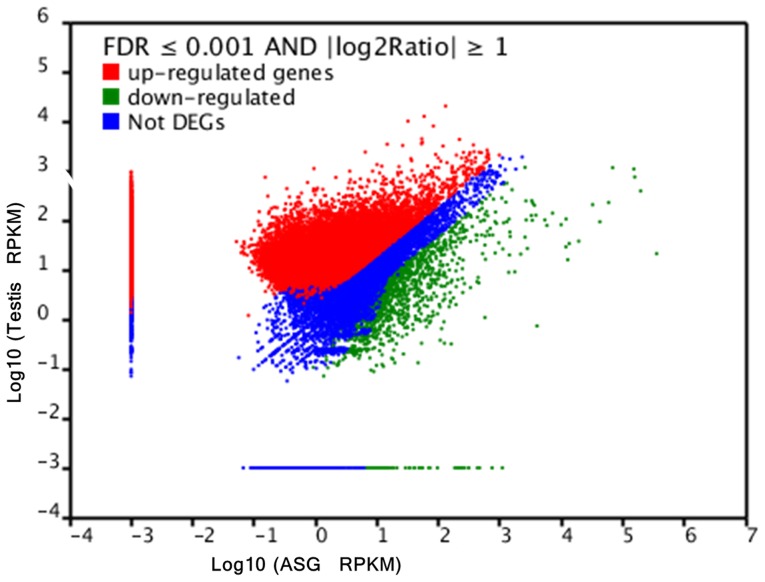
DEGs analysis of unigenes in the ASG and testis from *E. sinensis*.

Here, we investigated differentially expressed genes identified in our transcriptome analysis of ASG and testis tissues in *E. Sinensis*. Comparison of gene expression using DEGseq produced a total of 68,412 unigenes expressed in the testis at a significantly higher level than that in the ASG, and 5,174 unigenes were down regulated in testis compared with the ASG. On the other hand, of all the unigenes identified, 26,653 unigenes were expressed in the testis, but not expressed in the ASG, and 631 unigenes were expressed in the ASG, but not in the testis (Fig. 5 and Table S2).

## Discussion

Descriptive and quantitative transcriptome analyses are important for interpreting the functional elements of the genome and revealing the molecular constituents of cells and tissues. It is known that sperm function can be affected by ASG proteins, including the processes of capacitation and the acrosome reaction, as well as sperm motility, DNA integrity and interaction with the oocyte. Here we identified many ASG secreted proteins that function in the modification of sperm and in sperm maturation (Table 3), including P26h (L-xylulose reductase, unigene 4,288), BSPs (bovine seminal plasma protein, unigene 64,588 and unigene 69,768), fertilin (unigene 17,270 and unigene 27,136), ACE (Angiotensin converting enzyme, unigene 7,164 and unigene 21,069), GPX5 (glutathione peroxidase, epididymal secretory glutathione peroxidase, unigene15860), Spermadhesin-1 (Acidic seminal fluid protein, aSFP). The reproduction-related transcripts identified in the ASG and testis transcritptomes in *E. sinensis*, with a special focus on the process of sperm transit through the ASG and the proteins involved in sperm membrane modification will be discussed in detail in the following section.

### Proteins Involved in the Acrosome Reaction and Sperm-oocyte Interaction

P26h (L-xylulose reductase) catalyzes the NADPH-dependent reduction of several pentoses, tetroses, trioses, alpha-dicarbonyl compounds and L-xylulose. Functionally, P26h is involved in sperm–oocyte binding and its presence on sperm is an absolute prerequisite for fertilization [29]. Here we identified that Unigene4288 annotated as L-xylulose reductase (gi|229365856|gb|ACQ57908.1|; *Anoplopoma fimbria*), was expressed equally in the ASG (RPKM 27.5693) and testis (RPKM 19.8661). During epididymal transit, P26h accumulates on the acrosomal cap of spermatozoa. Moreover, P26h is found in epididymosomes and becomes GPI-anchored to the sperm surface of the acrosomal region during epididymal transit, via an as yet unknown mechanism. Similarly, PH-20 (Sperm adhesion molecule 1, SPAM1) is a glycoprotein synthesized by the principal cells that associates with epididymosomes [27]. PH-20 is located on the sperm surface and in the acrosome, where it is bound to the inner acrosomal membrane. PH-20 is a multifunctional protein which can serve as a hyaluronidase, a receptor for HA-induced cell signaling, and a receptor for ZP binding [30].

In the bull (*Bos taurus*), the seminal plasma contains a group of four closely related acidic proteins called Bovine seminal plasma protein (BSP) BSP-A1, BSP-A2, BSP-A3, and BSP-30-kDa that bind to sperm plasma membranes after ejaculation by specific interaction with phospholipids [31]. Here we identified two BSP unigenes (Unigene64588, Unigene69768) that were only expressed in testis (RPKM 6.192 and 4.7583 respectively). The BSP-A1 and BSP-A2 mixture referred to as PDC-109, constitutes the major protein fraction in bovine seminal plasma and contains two tandem repeat fibronectin type-II (Fn II) domains, each of which can bind to a choline phospholipid on the sperm plasma membrane by its specific interaction with the phosphorylcholine headgroup [32]. This interaction of PDC-109 with the sperm cell membrane results in an efflux of cholesterol and choline phospholipids, that appears to be important for capacitation.

The main changes in spermatozoa that occur during epididymal maturation are the ability to move, recognize and bind to the ZP, and to fuse with the plasma membrane of the oocyte. The cellular processes responsible for these new properties of the sperm are probably related to changes in the surface of the plasma membrane itself [33]. In all species studied to date, it appears that specific testicular sperm surface proteins are removed or processed further as gametes pass through the epididymis [34]. Disappearance of some of these proteins is clearly related to a specific proteolytic mechanism during epididymal transit. For most proteins, proteolysis induces either a change in their membrane domain distribution, as has been shown for fertilin/PH30, or a release of a cleaved protein in the epididymal medium, as is the case for ACE.

Among spermatozoa surface proteins, fertilin, a heterodimer complex composed of two integral membrane glycoproteins named α-fertilin (ADAM-1) and β-fertilin (ADAM-2), as well as several other ADAMs have been reported to be involved in sperm-oocyte recognition and in membrane fusion [35]. Here we identified five unigenes (Unigene17270, 18613, 62731, 71804, and 27136) annotated as fertilin α subunits but we did not identify β subunit in our annotation results. These unigenes all showed significantly higher expression in testis (shown in table 3). The fertilin α-β complex shares traits with certain viral adhesion/fusion proteins, notably the presence of a candidate fusion peptide [36]. Both proteins are members of the ADAM (a disintegrin and metalloprotease) domain protein family with sequences containing a pro-domain, a metalloprotease, a disintegrin and a cysteine-rich domain, EGF-like repeats, a transmembrane domain and a carboxy-terminal cytosolic tail [37]. The β subunit is present as a full length protein on the testicular sperm surface and is proteolytically transformed during the passage of spermatozoa through the caput [38], and cleaved into a 35 kDa form in spermatozoa [39]. This proteolytic processing results in the removal of the pro- and metalloprotease-like domains, with only the full or part of the disintegrin domain, the cysteine-rich domain, the EGF repeat, the transmembrane and the cytoplasmic domains remaining on the sperm cell. This processing also induces a relocation of the fertilin complex to a different plasma membrane domain on the mature spermatozoa [40].

### Proteins Associated with Sperm Motility

Little is known about the impact of ASG secretions on sperm motility. Semenogelins proteins are mainly synthesized in the seminal vesicles and are believed to have an inhibitory effect on the ability of sperm to move [41]. In contrast, another vesical product, fructose, has been reported to be the main source of energy for spermatozoa [42]. Enzymes in the polyol pathway, including aldose reductase and sorbitol dehydrogenase, have been identified in epididymosomes [43] and appear to be involved in a mechanism for modulating sperm motility during epididymal transit. Patel *et al*. demonstrated a positive correlation between seminal levels of fructose and the relative proportion of motile sperm [44], but other studies could not find such a correlation. Prostate-specific antigen has been reported to be involved in degradation of semenogelins and may therefore be expected to have a positive impact on sperm motility.

The Serpin (serine proteinase inhibitor) family is exclusively expressed in the rat cauda epididymis and up-regulated by androgens, and is secreted into the lumen to cover the sperm head [45]. Zhao *et al*. identified a Serpin family protein (As_SRP-1) that is secreted from spermatids during nematode *Ascaris suum* spermiogenesis (also called sperm activation) and showed that As_SRP-1 has two major functions. First, As_SRP-1 functions in *cis* to support major sperm protein-based cytoskeletal assembly in the spermatid that releases it, thereby facilitating sperm motility acquisition. Second, As_SRP-1 released from activated sperm inhibits in *trans* the activation of surrounding spermatids by inhibiting vas deferens-derived As_TRY-5, a trypsin-like serine protease necessary for sperm activation. Here we identified eleven unigenes, including: unigene12520, 13284, 13350, 14659, 1959, 24440, 31267, 32663, 3605, 5046 and 67995 that were annotated as serine proteinase inhibitors, which were differently expressed in ASG and testis (Table 3). On the other hand, vesicular exocytosis is necessary to create fertilization-competent sperm in many animal species, components released during this process could be more important modulators of the physiology and behavior of surrounding sperm than was previously appreciated [46].

Another factor that is implicated in the process of semen viscosity is zinc, which primarily originates from the prostate. This metal may be crucial for modulation of the three-dimensional structure of SgI and SgII rendering them more susceptible to proteolytic breakdown by seminal proteases [47]. Additionally, immunoglobulin G, which is a luminal protein in the epididymis, was present only in the epididymal fluid. Caveolin-1, previously found in prostasomes, which are membranous vesicles similar to epididymosomes, has also been detected in epididymal vesicles. Here we identified four unigenes annotated as Sperm protamine P1 (Unigene13877, 23278l, 29647 and 59678) with higher expression in testis, as sperm nuclear proteins, specifically protamine 2 which is a zinc-finger protein [48]. Interestingly, Zinc binding to the sperm nucleus varies proportionately with the zinc content of protamine 2 in sperm chromatin [49]. A previous report indicated that an abnormally high contribution of seminal vesicular fluid to sperm-rich fractions of the ejaculate creates a risk of depleting chromatin zinc and thereby impairing zinc-dependent chromatin stability [50]. Some of the enzymes important for the function of sperm are zinc metallo-enzymes and can thus become dysfunctional when zinc is deficient. One of these, sorbitol dehydrogenase (SoDH), utilizes sorbitol to provide sperm with fructose for energy, so that SoDH activity is correlated with sperm motility. Similarly, lactate dehydrogenase-X, another zinc metallo-enzyme, has also been reported to have some relationship with sperm motility [9]. To our knowledge, this is the first presentation of strong evidence for protamine gene expression in *E. senensis* testis and ASG.

### Proteins Involved in Protection of Sperm

We discussed the sperm protection mechanism in testis during spermatogenesis, but sperm have a long journey after leaving the testis and before it arrives at the oocyte for fertilization. Here we discuss the ASG proteins involved in protection of sperm during epididymal transition. GPX5 (Type 5 glutathione peroxidase, Epididymal secretory glutathione peroxidase) is a protein secreted by the caput epididymis in an epididymosome-associated form and is thought to be involved in protecting epididymal sperm against oxidative stress. Here we identified 10 epididymal secretory glutathione peroxidases (Table 3), most of them were higher expressed in testis and only unigene62805 and 7010 were slightly higher expressed in ASG. GPX5 protects cells and enzymes from oxidative damage, by catalyzing the reduction of hydrogen peroxide, lipid peroxides and organic hydroperoxide, by glutathione. It may constitute a glutathione peroxidase-like protective system against peroxide damage in sperm membrane lipids [51]. MIF (Macrophage migration inhibitory factor) is a protein found in rat, human and bovine epididymis and epididymal sperm [52]. MIF has been localized within apical protrusions of epithelial cells, in epididymosomes and associated with sperm in the epididymal lumen, thereby supporting the hypothesis of apocrine secretion mediated protein transfer via epididymosomes.

### Epididymosome Associated Transportation

Frenette and Sullivan proposed that the transfer of epididymal proteins to the sub-cellular compartments of the sperm is mediated by small membranous vesicles, known as epididymosomes [53]. Epididymosomes are electron dense vesicles secreted in an apocrine fashion that range between 50 and 500 nm in diameter. Proteins associated with epididymosomes are not processed through the endoplasmic reticulum and Golgi apparatus and are characterized by unusual glycosylation patterns. Epididymosomes are rich in cholesterol, with cholesterol: phospholipid ratios as high as 2, and have sphingomyelin as their major phospholipid. Epididymosomes contain lipid rafts, i.e. cholesterol and phospholipid-enriched microdomains [54]. These microdomains contain GPI-anchored and transmembrane proteins, as well as signaling molecules including protein tyrosine kinases, and may serve as a platform for transferring the proteins from the epididymal epithelium to a maturing sperm.

*In vitro* and *in vivo* studies have shown that these vesicles, which are present in the cauda epididymis and seminal plasma, transfer a number of proteins to sperm. Additionally, some of these proteins have been shown to be essential for sperm motility and fertility [55]. We observed two sizes of ASG vesicles referred to small and large vesicles that were thought to play a key role in *E. sinensis* similar to the described previously epididymosomes [20]. Furthermore, these vesicles, when observed under transmission and scanning electron microscopy, were thought to contain the enzymatic proteins or other activation factors required for spermatophore rupture, that were released immediately during homogenate isolation and processing. We hypothesize that in a natural mating context environmental parameters, including pH or spermatheca-produced factors, may induce the slow release of the vesicle contained proteins or factors [20]. In crabs, the ASG is an important component of the male reproductive system that opens at the junction of the seminal vesicle and ejaculatory duct. Secretions from the ASG, along with spermatophores from the seminal vesicle and spermatic fluid, enter the female spermatheca through the ejaculatory duct during mating. In Brachyura, spermatophores are delivered into the spermatheca of the female during mating and gradually are broken down to release free sperm into the spermatheca, thus facilitating spermatozoa and egg fusion to complete fertilization [56]. Given this important process, we focused on the ASG functions of spermatophore rupture and sperm maturation, in order to identify secreted proteins from the ASG that may be important in these processes.

### Important Signaling Pathways in the Testis and the ASG

We listed the top 30 pathways in Table 2, showing the number of differently expressed genes and all genes with pathway annotations. In our analysis, classes of genes that maintain relatively steady-state levels of gene expression included those controlling tissue remodeling, immunoregulation, cell-cycle progression, apoptosis, and growth. Development of reproductive tissue is a dynamic process involving coordinated interactions between regulators that assemble or edit the cellular constituents that support developing gametes [12]. The regulation of actin cytoskeleton, proteasome, adherens junction, cell cycle and SNARE interactions in vesicular transport pathways were identified and are all thought to be involved in spermatogenesis and sperm maturation.

The central importance of cAMP and PKA in driving tyrosine phosphorylation events associated with capacitation is well established [57]. Interestingly, the key components of the MAPK signaling pathway including MAP kinases, ERK1/2, and MEK, which were identified in our dataset, are implicated in various aspects of capacitation in human spermatozoa [58]. It is thought that sperm cells may also have unique signaling pathways. For example, the small GTPases in the Rop family are important for many aspects of cytoplasmic signaling. In sperm cells, some complicated signaling cascades may be simplified. For example, mitogen-activated protein kinase (MAPK) cascades are central to many signaling pathways in animals, and there is often cross talk between different members in different signaling pathways [59].

Cell cycle transitions may be controlled by regulation of the ubiquitin carrier and cyclin ligase destruction machinery. To date, our lab has reported detailed cDNA expression of some components of the ubiquitin-proteasome involved in reproduction in *E. sinensis*, including *Es*-UbS27, *Es*-UbL40, *Es*-SUMO, *Es*-Aos1/*Es*-Uba2 and *Es*-Ubc9, that were widely observed in the testis and ovary [60], [61]. We also identified the ubiquitin mediated proteolysis pathway in *E. sinensis* and believe such regulatory mechanisms are important for spermatogenesis (Table S3). Cyclin B transcripts are also present in the ASG and testis of *E. sinensis*, including unigene 11,678 (cyclin B, *Fenneropenaeus penicillatus*), unigene 17,729 (ovarian cyclin B, *E. sinensis*), and unigene 42,166 (cyclin B, *Litopenaeus vannamei*). It is therefore possible that similar posttranscriptional controls, as well as other regulatory constraints, are placed on the transcripts that encode the proteolytic machinery that selectively degrades cyclins. Taken together, the expression profile of this particular group of transcripts points to an interesting stage of testis and ASG development, that could lead to a greater understanding of the machinery involved in controlling mitosis and meiosis in the *E. sinensis* reproductive system.

Although we have only recently begun to study reproductive regulatory mechanisms at a molecular level in *E. sinensis*, the knowledge gained from these studies is proving insightful information. In future studies we will focus on sperm maturation and the role of ASG protein modification and transportation of sperm, and also we will focus on other important signaling pathways especially with respect to ASG factors that are associated with fertilization, potentially yielding key biomarkers of testicular and ASG function, that currently remain largely unknown in *E. sinensis*. In this respect, the results of the present study are the first to tackle a phenomenological description of this issue using a second generation sequencing method.

## Supporting Information

Table S1 **Sequences with significant BLAST matches against Nr database for** ***E. sinensis*** **.**
(XLS)

Table S2 **Differently expressed unigenes between ASG and testis during sexually mature stage in** ***E. sinensis*** **.**
(XLS)

Table S3 **KEGG pathway analysis for** ***E. sinensis*** **.**
(XLS)
